# Cyclic Nucleotide Monophosphates and Their Cyclases in Plant Signaling

**DOI:** 10.3389/fpls.2017.01704

**Published:** 2017-10-04

**Authors:** Chris Gehring, Ilona S. Turek

**Affiliations:** ^1^Biological and Environmental Science and Engineering, King Abdullah University of Science and Technology, Thuwal, Saudi Arabia; ^2^Department of Chemistry, Biology and Biotechnology, University of Perugia, Perugia, Italy; ^3^Leibniz Institute of Plant Biochemistry, Halle, Germany

**Keywords:** signaling, cGMP, cAMP, guanylate cyclase (GC) and adenylate cyclase (AC), receptor kinases, *Arabidopsis thaliana*

## Abstract

The cyclic nucleotide monophosphates (cNMPs), and notably 3′,5′-cyclic guanosine monophosphate (cGMP) and 3′,5′-cyclic adenosine monophosphate (cAMP) are now accepted as key signaling molecules in many processes in plants including growth and differentiation, photosynthesis, and biotic and abiotic defense. At the single molecule level, we are now beginning to understand how cNMPs modify specific target molecules such as cyclic nucleotide-gated channels, while at the systems level, a recent study of the Arabidopsis cNMP interactome has identified novel target molecules with specific cNMP-binding domains. A major advance came with the discovery and characterization of a steadily increasing number of guanylate cyclases (GCs) and adenylate cyclases (ACs). Several of the GCs are receptor kinases and include the brassinosteroid receptor, the phytosulfokine receptor, the Pep receptor, the plant natriuretic peptide receptor as well as a nitric oxide sensor. We foresee that in the near future many more molecular mechanisms and biological roles of GCs and ACs and their catalytic products will be discovered and further establish cNMPs as a key component of plant responses to the environment.

## Introduction

**FIGURE 1:**
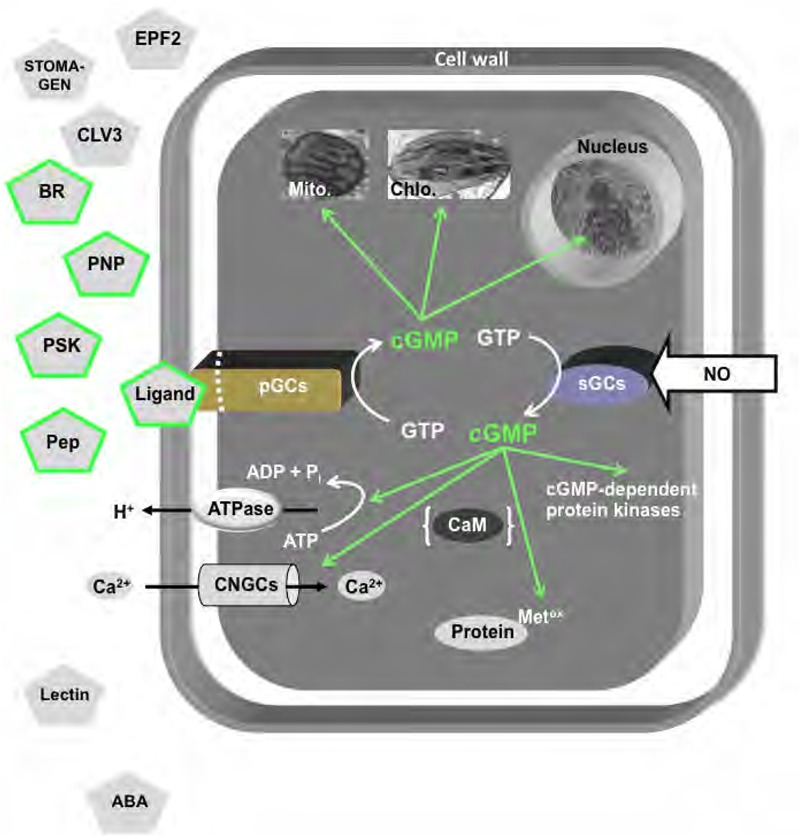
Overview of cGMP-dependent processes in plant cells. Particulate guanylate cyclases (pGCs) or soluble guanylate cyclases (sGCs) catalyze the reaction from GTP to cGMP that in turn modulates processes in the cell membrane, the cytosol, the mitochondria (Mito.), the chloroplast (Chlo.), or the nucleus. Proton transport is modulated by ATPases, while cation transport, including Ca^2+^ uptake, is enabled by cyclic nucleotide-gated channels (CNGCs) and calmodulin (Gonzalez-Fontes et al., 2014). Cyclic nucleotide-dependent processes also affect the phosphoproteome and other post-translational processes, including methionine oxidation. Established ligands of GC-coupled receptors are represented by pentagrams with green borders and include the PNP receptor (AtPNP-R1), the Pep receptor (AtPepR1), the brassinosteroid receptor (AtBRI-GC), and the phytosulfokine receptor (AtPSKR1). Candidate GC-coupled receptors include a Stomagen receptor, a CLAV3 receptor, an EPF2 receptor, and a lectin receptor.

There is a growing awareness that cyclic nucleotide monophosphates (cNMPs) and their cyclases are universal signaling molecules across the tree of life (Schaap, 2005). They are also key to many plant responses and the novel biological roles of these molecules and their mechanisms of action are discovered at an increasing rate (Meier et al., 2009; Wong et al., 2015; Marondedze et al., 2016a). Here, we review some of the early work on the detection of cNMPs in plants and the discovery of the cyclases that catalyze formation of 3′,5′-cyclic adenosine or guanosine monophosphate (cAMP or cGMP) and pyrophosphate from their corresponding nucleoside 5′-triphosphate. We will also review recent approaches to elucidate systems responses to cNMPs in plants (e.g., Isner et al., 2012; Marondedze et al., 2013; Alqurashi et al., 2016). A summary of cGMP-dependent processes is provided in **Figure 1**.

## A Brief Summary of the Roles of CAMP and CGMP in Plant Function

Despite the fact that cAMP and cGMP have been recognized as important second messengers in animals nearly half a century ago, the notion that the cNMPs signaling system is universal and operates in plants was a matter of debate until recently. The existence and physiological functions of cNMPs in higher plants have been questioned for long time mainly due to the fact that the cAMP and cGMP levels in plants appeared to be low (usually in the nanomolar range based on fresh weight) compared to those found in animals and lower eukaryotes (concentrations in the nano- to micromolar range) and often below the detection limits of analytical methods available in the past. Furthermore, reported amounts of cNMPs in different plants largely disregarded spatial and temporal variations of these secondary messengers. Arguably, the first convincing experimental evidence for the occurrence of cNMPs in higher plants came from a report that demonstrated changes in cNMPs levels during cell expansion and division in tobacco (*Nicotiana tabacum*) (Lundeen et al., 1973). Given the relatively low concentrations of cNMPs in plants, a second controversy concerned the biological significance of the effects exerted by non-physiological micromolar concentrations of exogenously applied cNMPs analogs on plant systems. However, numerous reports of nanomolar concentrations of cNMPs stimulating plant responses have since then overcome this point (e.g., Alqurashi et al., 2016; Marondedze et al., 2016a).

A growing body of evidence from plant electrophysiology and cell and molecular biology reveals that a considerable number of physiological processes in plants are correlated with alterations in cNMPs levels. Reports from several laboratories have shown direct effects of cNMPs on cation fluxes, including K^+^, Na^+^, and Ca^2+^, sparking interest in these molecules as potential regulators of ion and water homeostasis. Both cAMP and cGMP cause elevation of cytosolic Ca^2+^ concentration, involving internal and external Ca^2+^ stores, in tobacco (*Nicotiana plumbaginofolia*) protoplasts (Volotovski et al., 1998) and down-regulate Na^+^ influx and accumulation in Arabidopsis roots (Essah et al., 2003; Isner and Maathuis, 2011). Direct evidence for cAMP activation of hyperpolarization-activated Ca^2+^ channels in plasma membrane (PM) was provided in guard and mesophyll cells of *Arabidopsis thaliana* (Lemtiri-Chlieh and Berkowitz, 2004) and in pollen tube of Asian pear (*Pyrus pyrifolia*) (Wu et al., 2011). Cyclic GMP-dependent modulation of PM H^+^-ATPase activity, which indirectly provides a driving force for the cellular uptake of cations, particularly K^+^, and hence may significantly affect nutrient uptake and solute transport and could regulate cell volume, was reported in *Tradescantia multiflora* stem and leaf tissue (Suwastika and Gehring, 1999). K^+^ and Na^+^ fluxes modulated by cGMP by the activation of non-selective ion channels were observed in *Zea mays* roots (Pharmawati et al., 1999). Whole-cell patch-clamp current recordings in protoplasts from *Vicia faba* mesophyll cells revealed dose-dependent increases in the outward K^+^ current upon intracellular application of cAMP (Li et al., 1994), whereas cGMP was suggested to directly affect K^+^ fluxes in Arabidopsis guard cells (Pharmawati et al., 2001) and in intact tissue (Maathuis, 2006). Furthermore, both cNMPs were demonstrated to modulate K^+^ and Ca^2+^ flux responses to H_2_O_2_ in Arabidopsis roots (Ordoñez et al., 2014). In analogy to animal systems, many functions of plant cNMPs are primarily mediated through cyclic nucleotide-gated channels (CNGCs), many of which have already been demonstrated as critical for plant responses to both biotic and abiotic cues (Kaplan et al., 2007; Jha et al., 2016). One of the CNGCs characterized in Arabidopsis, namely AtCNGC4, that is permeable to both Na^+^ and K^+^, is activated by both cAMP and cGMP and has been implicated in plant responses to biotic stress (Balague et al., 2003).

Ample evidence for the role of both cAMP and cGMP in several physiological processes, including cell cycle progression as well as cell proliferation, growth and differentiation, organelle development, seed germination, and finally, plant growth and flowering have been accumulated. Early studies revealed changes in cGMP concentration that occur during cell volume increases and division in excised pith tissues of tobacco (Lundeen et al., 1973). Stringent control of intracellular cAMP levels was observed during progression through the cell cycle of the tobacco BY-2 cells, with cAMP peaks observed in S phase and, to lesser extent, in G_1_ phase (Ehsan et al., 1998). In contrast, reduced cAMP levels were noted at the beginning of G_1_ phase in arrested cells (Ehsan et al., 1999). cGMP was also implicated in chloroplast development (Bowler et al., 1994a), which could be inhibited by high level of cGMP (Bowler et al., 1994b; Wu et al., 1996). Immunochemical and cytoenzymological detection of cAMP and adenylate cyclase (AC) activity, respectively, was demonstrated in the developing tobacco chloroplasts (Witters et al., 2005), providing additional evidence for a general role of cNMPs in photosynthetic activity of higher plants, resembling well-characterized functions of these secondary messengers in lower plants. In addition to gibberellic acid (GA)-dependent elevation of cAMP concentration required for seeds germination, observed in common broomrape (*Orobanche minor*) (Uematsu et al., 2007) and phacelia (*Phacelia tanacetifolia*) seeds (Uematsu and Fukui, 2008), cGMP was identified as a positive regulator of Arabidopsis seed germination (Teng et al., 2010). In pollen, where tube grow and orientation (Prado et al., 2004) and targeting (Prado et al., 2008) are critical, the gaseous signaling molecule nitric oxide (NO) has a role too and as in other systems, cyclic nucleotides are implicated in downstream signals [for review see Domingos et al. (2015)]. In addition, cGMP was demonstrated to mediate adventitious root formation (Pagnussat et al., 2003), root gravitropism (Hu et al., 2005), and is vital for root growth and development by positive modulation of auxin-regulated signaling responses (Nan et al., 2014). Furthermore, cAMP was implicated in the regulation of symbiotic root nodule formation, with soybean (*Glycine max*) root nodules containing significant levels of cAMP, while low cAMP level was noted in leaves (Terakado et al., 1997), and low concentrations of this cNMP were demonstrated to induce self-incompatibility in lily (*Lilium longiflorum*) (Tezuka et al., 2007). Assigning guanylate cyclase (GC) activity to brassinosteroid receptor BRI1 (Kwezi et al., 2007) and phytosulfokine receptor PSKR1 (Kwezi et al., 2011) provided a link between cGMP signaling and regulation of multiple growth and developmental processes, including embryogenesis, cell proliferation and elongation, and vascular differentiation (Sauter, 2015; Wei and Li, 2016). Furthermore, cGMP has been also implicated in phytochrome-controlled induction of flowering of morning glory (*Pharbitis nil*) (Szmidt-Jaworska et al., 2008) and in programmed-cell death (PCD) (Clarke et al., 2000), again highlighting impact of cNMPs on nearly every aspect of plant life cycle.

It is evident that cNMPs are becoming increasingly recognized as modulators of plant metabolism and a recurrent theme that has emerged since the early descriptions of these fundamental secondary messengers is the importance of their interplay with plant hormones. For instance, GA treatment of cereal aleurone layers led to a transient increase in cGMP levels and the subsequent induction of α-amylase production that in turn causes the conversion of starch to sugar (Penson et al., 1996), whereas auxin and kinetin, which cause stomatal opening, also signal via this cNMP (Cousson and Vavasseur, 1998; Pharmawati et al., 2001). Elevated concentrations of cAMP coincide with the early stages of the response to phytoalexins and evoke production of an antifungal isocoumarin, 6-methoxymellein, in cultured carrot (*Daucus carota*) cells (Kurosaki et al., 1987; Kurosaki and Nishi, 1993). Furthermore, NO elicits many processes in plants [for review see Domingos et al. (2015)] and often does so together with cyclic nucleotides and notably cGMP, which in analogy to mammalian systems, has been strongly implicated in NO signaling in plants (Pfeiffer et al., 1994; Durner et al., 1998; Dubovskaya et al., 2011). Examples include calcium-dependent development of cell polarity in *Ceratopteris richardii* (Salmi et al., 2007), NO increases in mitochondrial respiration in Arabidopsis callus (Wang et al., 2010), and NO- and redox signaling in the phloem upon localized leaf wounding (Gaupels et al., 2016). Finally, cGMP mediates plant natriuretic peptides (PNPs) signal (Morse et al., 2004), pinpointing a general function of cNMPs in regulation of ion and solute homeostasis in plants.

Importantly, cNMPs are involved in perception of extracellular abiotic and biotic stimuli and subsequent amplification and transduction of the signals to corresponding responses. Numerous observations have provided incontrovertible evidence that cNMPs function in plant abiotic stress responses and this is often accomplished by regulation of the ion fluxes. Addition of membrane permeable cNMPs during growth assays of *A. thaliana* roots affected voltage-independent channels and reduced levels of Na^+^ accumulation, thus improving plant salinity tolerance (Maathuis and Sanders, 2001). A rapid (<1 min) time-dependent increases in cellular cGMP levels also occur after the salt and osmotic stress treatments (Donaldson et al., 2004) and CNGC19 and CNGC20 were speculated to presumably be involved in these early responses (Kugler et al., 2009). Elevated cGMP was also noted in late (>2 h) response to ozone (Pasqualini et al., 2009), whereas increased cGMP level upon a 30 min-long heat stress was associated with CNGC16 (Tunc-Ozdemir et al., 2013). These increases point to physiological roles of cNMPs in abiotic stress responses. It is noteworthy that proteomic study also suggested that cAMP functions in responses to environmental threats, such as temperature, as well as in light signaling and regulation of photosynthesis (Thomas et al., 2013). Early studies revealed considerable fluctuations of cGMP levels occurring in response to light (Brown et al., 1989; Bowler et al., 1994b), and a direct effect of cGMP concentrations on the phytochrome signal transduction was elucidated by investigating *aurea* mutant of tomato that is deficient in phytochrome A, a photoreceptor responsible for regulating many morphogenesis responses, including flowering, seed germination, and diurnal rhythms; cGMP not only triggered the production of photoprotective compounds anthocyanins, but in combination with Ca^2+^ induced development of fully mature chloroplasts containing all the photosynthetic machinery (Bowler et al., 1994a). In line with these observations, recently reported interactors of cNMPs include several enzymes involved in Calvin cycle (Donaldson et al., 2016), implying specific roles of cNMPs in photosynthesis.

An impact of cNMPs on gas exchange can also be assumed based on early observations describing effect of cAMP analogs in stomata opening in *V. faba* (Curvetto et al., 1994), while cGMP analog was reported to induce stomata opening in a number of plants, including Arabidopsis (Pharmawati et al., 1998, 2001); this occurs downstream of abscisic acid (ABA)-induced changes in H_2_O_2_ and NO (Dubovskaya et al., 2011). Consistent with the notion of cNMPs involvement in photorespiration, several photorespiratory enzymes were observed among proteins identified as cAMP or cGMP interactors (Donaldson et al., 2016). Furthermore, differential effects of cGMP and 8-nitro-cGMP on stomata opening (Joudoi et al., 2013) were reported, whereas, similarly to cGMP, cyclic adenosine 5′-diphosphoribose (cADPR) was shown to positively function in ABA and methyl jasmonate-induced stomatal closure in Arabidopsis (Hossain et al., 2014). Since stomata can be considered a frontline of plant interaction with the environment, their closure can be induced by adverse conditions, including not only drought and salt, but also pathogen attack, to reduce water loss and cell dehydration. Thus, function of cNMPs in plant responses to biotic stress appears plausible.

During the past two decades, the role of endogenous cAMP and cGMP in plant defense responses gained more recognition. cGMP was also reported to cause transcript increases of the defense-related genes encoding pathogenesis-related 1 and phenylalanine ammonia lyase proteins in tobacco (Durner et al., 1998) and to participate in signal transduction pathways activated by a group of danger-associated molecular patterns (DAMPs), such as AtPeps (Qi et al., 2010). NO has also a central role in defense against pathogens and the salicylic acid pathogen defense response pathways do not just include NO but also cGMP (Klessig et al., 2000) and this holds true for NO-triggered PCD (Clarke et al., 2000). In accordance with this finding, challenging *A. thaliana* with avirulent strain of *Pseudomonas syringae* results in cGMP accumulation (Meier et al., 2009). More recently it has been demonstrated that infection of *A. thaliana* with avirulent pathogens (and not avirulent strains) can cause a biphasic increase of cGMP downstream of NO. However, constitutive cGMP accumulation can compromise systemic acquired resistance (Hussain et al., 2016) and this would suggest that “swamping” the cell with a signaling molecule can cause disfunction. Accumulation of cGMP was also demonstrated in extrafascicular phloem, a defensive structure against herbivorous animals, in leaf wounded pumpkin (*Cucurbita maxima*) (Gaupels et al., 2016). Similarly, pathogen attack and mechanical wounding elevated cGMP content in *Hippeastrum hybrid* (Swiezawska et al., 2015). On the other hand, cAMP, apart from being implicated in activation of phytoalexin synthesis in sweet potato (*Ipomoea batatas*) (Oguni et al., 1976) and in early signaling events in the apoplastic oxidative burst (Bindschedler et al., 2001), was also reported to be elevated at the infection site initiation in pathogen-related cytosolic Ca^2+^ signaling (Ma et al., 2009). Moreover, production of elevated concentrations of cAMP by the administration of elicitors has been demonstrated in French bean (*Phaseolus vulgaris*) (Bolwell, 1992), carrot (*D. carota*) (Kurosaki and Nishi, 1993), alfalfa (*Medicago sativa*) exposed to *Verticillium alboatrum* glycoprotein (Cooke et al., 1994), Mexican cypress (*Cupressus lusitanica*) cell culture treated with yeast oligosaccharides (Zhao et al., 2004), and Arabidopsis treated with *Verticillium dahliae* toxins, resulting in improved disease resistance of the plants (Jiang et al., 2005). It is noteworthy that many signaling cascades generated in response to biotic stresses not only employ cNMPs, but also critically depend on CNGCs. For instance, AtCNGC4, activated by cAMP and cGMP, is induced as a result of pathogen infection and certain pathogen-related signals (Balague et al., 2003), whereas CNGC11 and CNGC12 activate multiple pathogen resistance responses and act as positive mediators of plant resistance against avirulent isolate of oomycete *Hyaloperonospora parasitica* (Yoshioka et al., 2006). Interestingly enough, recently several other CNGCs, including CNGC2 and CNGC4, have also been associated with plant responses to biotic stresses (Ali et al., 2007; Chin et al., 2013). In Arabidopsis, AtCNGC2 has recently been demonstrated to induce apoplastic Ca^2+^ influx in response to jasmonic acid (JA) and thus linking JA-induced cAMP increases to Ca^2+^-dependent downstream signaling (Lu et al., 2016). The rapid (<5 min) JA-dependent cytosolic cAMP increases and concomitant increase in cytosolic Ca^2+^ are indeed consistent with cAMP being an AtCNGC2 ligand (Lu et al., 2016). It is noteworthy, that another recent report suggests that AtCNGC2 delivers Ca^2+^ from veins into leaf cells thereby preventing apoplastic Ca^2+^ accumulation but does not seem to have a direct influence on the hypersensitive response (Wang et al., 2017).

Furthermore, wounding stress was shown to lead to a rapid approximately five-fold increase in concentration of the non-canonical 2′,3′-isomers of cAMP and cGMP in *A. thaliana* leaves (Van Damme et al., 2014). These reports, taken together, strongly argue that both the host–pathogen recognition and the downstream processes are transduced by complex cNMP signatures and often include regulation of CNGCs. The role of cNMPs in both abiotic and biotic stress responses does not come as a surprise considering the fact that in biotic interactions pathogens modulate plant host ion and water homeostasis, inflicting abiotic stress conditions, to the detriment of the host (Gottig et al., 2008).

Since the low concentrations of cNMPs in higher plants definitely constituted a major problem in cNMP research in this system, it can be noteworthy to emphasize the impact of technological progress in the development of methods for extraction, purification, detection, and quantitation of cNMPs, with a very low limit of detection, on the advancement in investigation of plant cNMPs signaling witnessed over last few decades. Initial methods of cNMPs separation and detection, such as radio-immune assays, characterized by a femtomole dynamic range, suffered from lack of accuracy due to co-elution of interfering plant metabolites, including derivatives of cNMPs and hence significantly compromising the data. This problem was largely overcome due to the use of anti-cAMP or anti-cGMP antibodies in an immunopurification step, allowing more powerful sample clean-up. In the 1980s, chromatographic analyses in plant cNMPs analysis, including paper and thin-layer chromatographies, were replaced by high-performance liquid chromatography (LC) by virtue of its capability to separate the 3′,5′-cyclic nucleotides and their 2′,3′-isomers (Brown, 1983). Nevertheless, unambiguous demonstration of the identity of the putative cNMPs obtained in cell extracts and as the product of incubations with AC and guanylyl cyclase (GC) was accomplished by the use of physical techniques, mass spectrometry (MS) in particular. The fast atom bombardment (FAB) collision-induced dissociation (CID) tandem MS (MS/MS) not only allowed the unequivocal identification of cAMP and cGMP, but also demonstrated natural occurrence of cytidine, inosine, uridine, and 2′-deoxythymidine 3′,5′-cyclic monophosphate (cCMP, cIMP, cUMP, and cdTMP, respectively) in pea (*Pisum sativum*) (Newton et al., 1991) in the early 1990s. Due to its greater sensitivity, LC–electrospray ionization (ESI)–MS/MS set-up, which decreased detection limits to femtomole level, gradually replaced FAB–MS/MS and is successfully used in cNMP detection and quantification studies until now, providing an excellent platform for large-scale investigation of cNMPs interactors and system-level analyses of proteins that are differentially expressed and/or post-translationally modified in a cNMP-dependent manner. Further advancement of separation techniques enabled analysis of 2′,3′-cNMPs in plants (Pabst et al., 2010) and facilitated studies on physiological relevance of both the canonical and non-canonical cNMPs in plants over the last decade (Van Damme et al., 2014).

The analytical chemistry methods developed for determination of cNMPs concentrations with fine enough spatial and temporal resolution that provides confidence in the biological significance of the results, although powerful, are labor intensive and costly in terms of infrastructure, require high levels of expertise and large amount of tissue. The enzyme immunoassays (EIAs), tailored for animal cells, used to determine contents of cAMP or cGMP in analyzed plant samples suffered greatly from lack of specificity and selectivity, often leading to inconsistent results (Newton and Smith, 2004). Newer enzyme-linked immunosorbent assays (ELISAs) are more specific for cNMPs but may under-report amounts of cNMPs present in plant cells (Meier et al., 2010), whereas a recently improved EIA method for cAMP detection in plant tissue was promised to be 10 times more sensitive than previously used methods (Lomovatskaya et al., 2011). The advent of biosensor-based techniques in cell biology, and in particular reporter systems used to record cNMP levels non-destructively in real time and with relatively high spatial and temporal resolutions, was a significant step forward in plant cNMPs research. However, in plant research, we are still quite distance away from the development and application of high-resolution sensing, e.g., with cytosolic Förster resonance energy transfer-based cGMP biosensors (Gotz et al., 2014) that are currently applied in medial research. Another obstacle that is intrinsic to plant work is the very small cytosolic compartment of cells where by far the biggest volume is taken up by the vacuole which is almost certainly not the place where cNMP-dependent signaling has a key role. Still, owing to the ease of use, the currently available plant reporters have significantly contributed to establishing the physiological functions of cNMPs in plants and these reporters also offer an affordable alternative to the advanced techniques of analytical chemistry.

In brief, firstly, the imaging of cAMP cellular distribution and quantification was reported in living pollen tubes (Moutinho et al., 2001) and it was achieved by microinjecting a cAMP fluorosensor. The pollen tube was chosen to investigate the role of cAMP not least because it is a large single cell that lends itself to microinjecting a fluorescent sensor dye into its cytoplasm. Secondly, reliable quantification of cytoplasmic cGMP concentrations in plant cells transiently and stably transformed with δ-FlincG in response to various external stimuli has also been reported (Isner and Maathuis, 2011). The reported δ-FlincG dissociation constant for cGMP was approximately 200 nM and was reported to result in a dynamic range spanning from approximately 20 nM to 2 μM. Thirdly, the development of luciferase-based promoter reporter systems. The principle here is that the promoter of a cGMP-inducible gene [e.g., OLIGOPEPTIDE TRANSPORTER X (OPTX)] is fused to a luciferase reporter gene (Wheeler et al., 2013) and luciferase activity is a proxy for cGMP and can be calibrated.

In summary, the development and application of sophisticated methods in the fields of immunocytochemistry, electrophysiology, and separation technologies combined with MS have assisted in the in-depth characterization of components of the plant cNMP-dependent signalosome.

## The Discovery of the First GCS in Higher Plants

**FIGURE 2:**
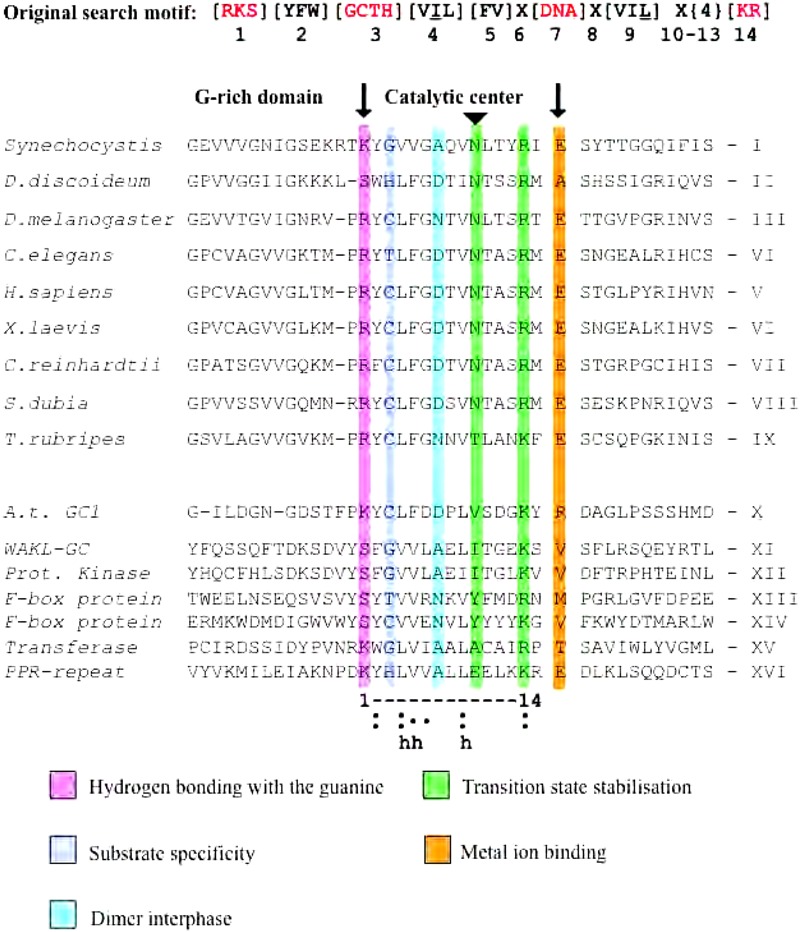
Alignment of GC catalytic domains led to the original GC search. Alignment of catalytic centers of GC delineated by two solid arrows. The deduced 14 amino acid-long search motif is in bold, and substitutions are in square brackets; X represents any amino acid, and curly brackets define the number of amino acids. Red amino acids are functionally assigned residues of the catalytic center and the underlined amino acids in positions 4 and 9 are the third branched aliphatic amino acid not appearing in the alignment. The C-terminal residue implied in Mg^2+^-binding or Mn^2+^-binding was not included in the original search motif. The letter “h” stands for hydrophobic residues forming the hydrophobic pocket.

Despite the fact that by the year 2000 the presence of cNMPs in higher plants was established beyond reasonable doubt and numerous cAMP- and cGMP-dependent processes have been reported, the cyclases responsible for the formation of cNMPs have remained elusive. While basic local alignment search tool (BLAST) searches with annotated GCs from lower eukaryotes and animals did return candidate GC in cyanobacteria perhaps surprisingly, when ACs and GCs from lower or higher eukaryotes were used to query the newly available *A. thaliana* genome no plausible plant candidates were returned (Ludidi and Gehring, 2003). This suggested that plant cyclases had lost ancestral cyclases domains and evolved new ones, or more likely, that plant cyclases had evolved in such a way that only the key catalytic residues remained conserved and that those few key amino acid (aa) residues where beyond the detection limit of BLAST searches. In order to test the second hypothesis, we firstly determined the key aa residues with annotated roles in the catalytic process (Liu et al., 1997; Tucker et al., 1998). These have in part been determined based on the crystal structure of the rat type II AC C2 catalytic domain that was in turn used to homology model a mammalian AC C1–C2 domain pair, a homodimeric AC of *Dictyostelium discoideum*, a heterodimeric soluble GC (sGC), and a homodimeric membrane GC. These authors also docked Mg^2+^-complexed adenosine triphosphate (ATP) or guanosine triphosphate (GTP) to the active site to inspect stereo-chemical constraints of the resulting conformation (Liu et al., 1997). The authors report that their “models are consistent with the activities of seven mutants” in the catalytic site where an aspartic acid (D) and glutamine (Q) of type I ACs can coordinate the Mg^2+^ ion. They also show that mutating D310 residue to serine (S) and (D) 310 to alanine (A) decreases the reduced *V*_max_ of the reaction as well as altering [Mg^2+^] dependence. Furthermore, it was proposed that the purine moieties bind in hydrophobic pockets and that specificity is due to a lysine (K) and D in AC, and a glutamic acid (E), an arginine (R), and a cysteine (C) in GCs. It was also predicted that an asparagine (N)–R pair would stabilize the transition state. With these structural parameters in mind, we then inspected alignments of known catalytic centers of different types of nucleotide cyclases (McCue et al., 2000) before aligning catalytic centers of annotated, and in some cases, experimentally confirmed nucleotide cyclases in lower and higher eukaryotes (Ludidi and Gehring, 2003). From this alignment, we extracted a 14 aa-long core catalytic center search motif (**Figure 2**) and when used to query the Arabidopsis proteome, it returned seven candidate GCs, including AtCG1 (At5g05930) and AtWAKL10 (At1g79680).

A recombinant AtCG1 was first to be tested experimentally and shown to be capable to generate cGMP for GTP *in vitro* in the presence of Mg^2+^. AtGC1 is a dual-domain 274 aa-long sGC with the catalytic center on the very N-terminus, but contrary to sGCs in animals, AtGC1 is not sensitive to NO and therefore represented a new type of sGC. The second domain has been predicted to be a peptidase family C39-like domain (IPR005074) typical for antibiotic bacteriocin-processing endopeptidases from bacteria where the cleavage is mediated by its ATP-binding cassette (ABC) transporter domain as part of the secretion process and this is supported by fold studies suggesting that AtGC1 is a member of a novel eukaryotic C proteinase family that might function as a protease (Ginalski and Zemojtel, 2004). While preliminary studies have confirmed the protease activity, the interrelationship between the GC activity and the protease activity as well as the biological function(s) has remained elusive.

We also tested AtWAKL1 that is a receptor type wall associated kinase-like molecule that can also generate cGMP *in vitro* as well as having kinase activity (Meier et al., 2010). Furthermore, a co-expression and stimulus-specific expression analysis of AtWAKL10 has suggested that it is consistently co-expressed with extensively characterized pathogen defense-related genes and, much like these genes, is induced early and pronouncedly in responses to both a range of pathogens (including *Botrytis cinerea* and *P. syringae*) and their elicitors (Meier et al., 2010).

## Expanding the Search and Discovery of Novel Cyclase in Higher Plants

How many mononucleotide cyclases (MNCs; ACs and GCs) are there in the proteomes of higher plants? This question has remained unsolved, but several lines of evidence suggest that the number may be considerably higher than previously suspected and may in fact be >100. Firstly, we noted that *Chlamydomonas reinhardtii*, a unicellular green alga, contains >100 MNCs annotated on the basis of sequence homology with predicted and/or experimentally confirmed ACs and GCs. These MNCs come in >20 different domain combinations and configurations with 13 different partners including Heme NO/OXygen (H-NOX), periplasmic-binding protein, GAF-like and protein kinase-like domain, ATPase domain of HSP90, RNI-like, ribonuclease-H, duplicated hybrid motif, CheY-like, family AG protein-coupled receptors, homodimeric domain of SIG, SAM/pointed domain, and C proteinase (Meier et al., 2007). Still, while the dependence on cNMPs of biological processes such as sexual signaling by gametes (Pasquale and Goodenough, 1987) and nitrate assimilation (de Montaigu et al., 2010) have been described, the cyclases at the source of the signaling pathway have remained elusive.

Secondly, given the number and diverse nature of cNMP-dependent processes such as stomatal guard cell movement (Curvetto et al., 1994; Pharmawati et al., 1998; Joudoi et al., 2013), responses to light and temperature (Brown et al., 1989; Bowler et al., 1994b; Thomas et al., 2013), responses to pathogens (Bindschedler et al., 2001; Meier et al., 2009; Gaupels et al., 2016; Hussain et al., 2016) and Pep peptides (Qi et al., 2010), responses to NO and ozone (Clarke et al., 2000; Pasqualini et al., 2009), cation transport (Hoshi, 1995; Pharmawati et al., 1999; Maathuis, 2006; Ordoñez et al., 2014), the modulation of CNGCs (Ali et al., 2007; Zelman et al., 2012; Chin et al., 2013; Tunc-Ozdemir et al., 2013), and the specific cGMP-dependent protein phosphorylation (Marondedze et al., 2016a) and methionine oxidation (Marondedze et al., 2013), it seems highly unlikely that only a very small number of ACs and CGs is responsible for all of these complex reactions. It appears more likely that higher plants, much like Chlamydomonas, harbor a large number of diverse MNCs that are capable to deliver distinct spatial, temporal, and stimulus-specific transient signals.

Thirdly, Chlamydomonas MNCs do not seem to be closely related to proteins in *A. thaliana*. The lowest *e*-value of a Chlamydomonas MNC protein (or protein fragment) compared against Arabidopsis proteins is 0.009 and this protein is a putative ethylene-responsive DEAD box RNA helicase (At5G63120). Another three Chlamydomonas MNC proteins have BLAST *e*-values < 0.05 but >0.01. This makes the evolution of MNC from Chlamydomonas to Arabidopsis at best a matter of speculation only (Meier et al., 2007).

Given these considerations, we hypothesize that higher plants are likely to contain a large number of MNCs and that the most promising way forward was to use catalytic center motifs as a means to identify them. Modifications of the motif would be based on rational considerations and in some cases tested by site-directed mutagenesis of experimentally proven cyclases (Kwezi et al., 2007). This led to a derived more relaxed search motif ([R]X{5,20}[RKS][YFW][GCTH][VIL][FV]X{3}[VIL]X{4}[KR]X {1,2}[D]) that occurs in 27 *A. thaliana* proteins of which AtBRI1 (At4g39400), the receptor for the brassinosteroids, a class of plant hormones, was the first to be tested (Kwezi et al., 2007). AtBRI1 is a leucine-rich repeat receptor-like kinase (LRR-RLK) from which we have cloned and expressed a 114 aa-long recombinant protein (AtBRI1-GC) that harbors the GC domain. This domain can convert GTP to cGMP *in vitro*, a finding that suggested that AtBRI1 may belong to a novel class of GCs that contains both a cytosolic kinase and GC domain and we have subsequently shown that the GC activity of AtBRI1 enables brassinosteroid signaling by rapidly phosphorylating the downstream brassinosteroid signaling kinase 1 (BSK1) (Wheeler et al., 2017). Incidentally, AtBRI1 and other plant LRR-RLKs have a domain organization that is not dissimilar to that of animal atrial natriuretic peptide (ANP) receptors, NPR1 and NPR2 (Singh et al., 1988; Chinkers et al., 1989). Since ANPs bind specifically to PMs and elicit physiological responses (e.g., by stomatal opening) (Gehring et al., 1996) and plants also contain highly biologically active compounds that turned out to be functional homologs of ANPs, that also cause cGMP transients [for review see Gehring and Irving (2003) and Gehring and Irving (2013)], it was not entirely surprising when a recent report found and described the PNP receptor and showed that it contains a cytosolic GC domain that is activated by the ligand PNP (Turek and Gehring, 2016).

A second candidate tested in this group is AtPepR1 (At1g73080). It is a receptor for a family of peptide signaling molecules (AtPeps) that act as DAMPs in pathogen defense signaling cascades in plants (Qi et al., 2010). We showed that this LRR-RLK also has GC activity and that AtPep-dependent expression of biotic stress defense genes (*PDF1.2, MPK3*, and *WRKY33*) is linked to Ca^2+^ signaling pathway that in turn are associated with AtPeps and Pep receptors.

Interestingly, these 27 Arabidopsis proteins are highly significantly (*p*-value < 1e^-5^) enriched for the gene ontology (GO) categories of “phosphorus metabolic processes,” “protein metabolic process,” “cellular macromolecular metabolic process,” and “biopolymer metabolic process.” Furthermore, several proteins are annotated as LRR-RLKs and they include AtBRI1 (brassinosteroid insensitive 1; At4g39400), which is the receptor for brassinosteroids. What appears quite unusual about the domain architecture of this trans-membrane receptor kinase is that the GC domain is encapsulated in the cytoplasmic kinase domain, begging question of the evolutionary and functional relationship of these two domains. We have subsequently shown that AtBRI1 has GC activity (Kwezi et al., 2007) and that cGMP enables brassinosteroid signaling by rapidly phosphorylating the downstream BSK1 (Wheeler et al., 2017).

Also included in the GC candidate list are three receptor kinases of the ERECTA family: At2g26330 ERECTA (ER), At5g62230 ERECTA-like1 (ERL1), and At5g07180 (ERL2) [for review see Ho et al. (2016), Ikematsu et al. (2017), and Lin et al. (2017)]. Arabidopsis *erecta* (*er*) mutants show altered organ shape and the ecotype Landsberg (Ler) is typified by compact inflorescences with flowers clustering at the top and also have round leaves with short petioles and siliques (Bowman, 1993). Furthermore, they include photomorphogenesis, phytohormone biosynthesis and signal transduction, and flower organ identity mutants. More recently, many of theses mutants have been identified as LRR-RLKs (Torii et al., 1996). At2g26330 (*ERECTA*) is a quantitative trait locus for transpirational efficiency due, in part, to its influence on epidermal and mesophyll development including stomatal density and porosity of leaves, and it has been shown to be required for callose deposition upon infection (Sanchez-Rodriguez et al., 2009) and implicated in the resistance to the soil borne bacterium *Ralstonia solanacearum*, the necrotrophic fungus *Plectosphaerella cucumerina*, and oomycetes from *Pythium irregulare* (Godiard et al., 2003; Llorente et al., 2005; Adie et al., 2007) as well as in thermotolerance (Shen et al., 2015). The second candidate, At5g62230 (*ERL1*), also encodes an RLK that, together with ER and ERL2, has a key role in the fate of protodermal cells to divide proliferatively and produce pavement cells or, alternatively, to undergo asymmetric divisions that will eventually form stomatal complexes. The third candidate, the RLK At5g07180 (*ERL2*), determines whether protodermal cells divide proliferatively to produce pavement cells or divide asymmetrically to generate stomatal complexes as well as for maintaining stomatal stem cell activity in order to prevent terminal differentiation into the guard mother cell (Staff et al., 2012). *In silico* identification of the candidate GC catalytic centers in these proteins may suggest that cGMP acts as a messenger in the developmental programming of stomatal formation. However, the biochemical proof of GC activity of these RLKs still awaits confirmation.

Furthermore, CLAVATA1 (CLV1; At4g20270) is another annotated LRR-RLK. It is expressed in the shoot apical meristem where stem cells are at their undifferentiated stage by inhibiting WUSCHEL (WUS) and FANTASTIC FOUR (FAF). *WUS* and *FAF2* expression is inhibited by the CLV3 peptide in wild-type plants and impaired with Ca^2+^ channel blockers and GC inhibitors. This inhibition was also observed in the DEFENSE NO DEATH 1 (*dnd1*) and 2 (*dnd2*) mutants that lack the CNGC 2 and 4, respectively (Chou et al., 2016). This may suggest that when CLV3 binds to the CLV1 receptor in the meristem, an increase in cGMP results and that may in turn activate CNGC2 channels and consequently increase cytoplasmic Ca^2+^. Elevated Ca^2+^ concentrations have been reported to cause altered expression of *WUS* and *FAF2* and eventually change the fate of the meristem cell (Isner and Maathuis, 2016). Perception of CLV3 peptide by CLV1 activates mitogen-activated protein kinases (MPK) MPK3 and MPK6 signaling (Betsuyaku et al., 2011; Lee et al., 2011), that in turn is involved in mediating multiple developmental and stress responses, including responses to biotic cues. Interestingly enough, involvement of CLV1 in signaling and/or perception of CLV3/endosperm surrounding region (CLE)-like effector proteins secreted by plant-parasitic beet cyst nematodes was revealed since infection with *Heterodera schachtii* is reduced on *clv1* mutant and *CLV1* expression was induced in the nematode feeding sites (Replogle et al., 2013). More recent study investigated the function of both nematode A- and B-type CLE peptides in the regulation of cell proliferation at site of feeding, linking the function of CLE receptors, including CLV1 to the modulation of the vascular stem cell pathways (Guo et al., 2017).

In order to identify additional candidate GCs we have further modified the search motif ([R]X{5,40}[RKS][YFW] [GCTH][VIL][FV]X{3}[VIL]X{4}[KR]X{2,3}[D]) and retrieved 107 candidates and subjected them to GO search. Of the significantly enriched terms, we chose these in the category of “trans-membrane receptor protein tyrosine kinase signaling pathway” [*p*-value: 2.7e^-10^; false discovery rate (FDR): 2.1e^-09^] and identified four candidates: At2g02220, phytosulfokine receptor 1 (AtPSKR1); At3g13065, strubbelig-receptor family 4 protein (SRF4); At5g58150, an uncharacterized LRR-RLK; and At5g65710, the HAESA-Like 2, HSL2. Of those, AtPSKR1 is perhaps the best studied GC. Not only was it shown that the recombinant complete kinase (cytoplasmic) domain of AtPSKR1 has serine/threonine kinase activity (approximate *K*_m_ of 7.5 M and *V*_max_ of 1800 nmol min^-1^ mg^-1^ protein), but also that this domain has a GC activity *in vitro*. It was further demonstrated that over-expression of the full-length AtPSKR1 receptor in *A. thaliana* leaf protoplasts caused a >20-fold increase in endogenous cGMP levels and, importantly, that the addition of the active sulfonated ligand, PSK, induces rapid increases in cGMP levels in protoplasts (Kwezi et al., 2011). This is entirely consistent with the idea that the cytosolic GC domain of the receptor is activated by the ligand and enables to downstream signal via cGMP and suggests that the receptor GCs where the catalytic GC center is embedded in functional kinases are in fact dual-functioning – moonlighting – enzymes (Irving et al., 2012). The arising question of the functional relationship of the two domains was also addressed and it was demonstrated firstly, that Ca^2+^ is the switch between the kinase and the cyclase activity in these moonlighting enzymes (Muleya et al., 2014) and secondly, that specific auto-phosphorylation and dimerization are essential mechanisms for ligand-mediated catalysis and signaling (Muleya et al., 2016). The remaining three candidates: SRF4, the un-annotated LRR-RLK, and HSL2 remain biochemically uncharacterized. SRF4 is likely to operate as a “positive regulator” of leaf size (Eyüboglu et al., 2007) but it is also transcriptionally activated by, e.g., ABA, drought, and the plant pathogen *Golovinomyces cichoracearum.* HSL2 participates in abscission and in particular floral abscission (Patharkar and Walker, 2015) and is induced by, e.g., ABA and the root-knot nematode *Meloidogyne incognita*.

## Soluble GCS and NO Sensing

Nitric oxide is a gaseous reactive oxygen species (ROS) that has evolved as a signaling hormone in many physiological processes in animals and plants, and in animal systems NO-dependent sGCs have long been known and are well characterized (Knowles et al., 1989; Denninger and Marletta, 1999). In plants, NO has been demonstrated to operate as regulator of development functioning as a signaling molecule at each step of the life cycle. NO has also been implicated in biotic and abiotic stress signaling [for review see Domingos et al. (2015)]. However, despite the multitude of plant responses to NO, many of them concomitant with cGMP increases, there have until recently been no sensor molecules discovered that can link NO to the GC activation.

In animal systems, NO binds to the heme group localized at a domain termed H-NOX, and this binding activates the GC catalytic center leading to the generation of cGMP from GTP (Palmer et al., 1987; Bellamy et al., 2002). Mutational and structural studies have confirmed the histidine (H) residue as a proximal ligand for the docking to the iron core of the heme porphyrin moiety (Wedel et al., 1994; Zhao et al., 1998) and the binding results in a 5-coordinate complex (Stone et al., 1995) that becomes a nitrosyl complex when bound to NO (Stone and Marletta, 1994). It can then sever the proximal H–Fe bond and lead to the subsequent displacement of the heme moiety (Dai et al., 2012). Given the chemistry of the structurally highly conserved H-NOX reaction center (Boon et al., 2005), we have again built a consensus motif search term (HX{12}PX{14,16}YXSXR) and retrieved four candidate molecules presumably capable of NO binding (At1g62580, At4g01160, At5g19160, and At5g57690). One of the candidate molecules (AtNOGC1, At1g62580) also contains the key residues required for GC catalytic activity ([RKS]X[GCTHS]X{9,10}[KR]) and is annotated as a flavin monooxygenase (Mulaudzi et al., 2011). Electrochemistry revealed that the recombinant binds NO and has higher affinity for NO than for O_2_ and that AtNOGC1 can generate cGMP from GTP *in vitro* in a NO- and time-dependent manner. More recently, it was also demonstrated that in NO-dependent stomatal closing nitration of cGMP (8-nitro-cGMP) is key and that in the *Atnogc1* mutant neither cGMP nor the cell permeant 8-bromo-cGMP induce stomatal closure (Joudoi et al., 2013) making AtNOGC1 an essential component in this signaling pathway.

## Finding ACS in Higher Plants

Establishing the presence and functions of cAMP and ACs in plants and higher plants in particular has been a long and at times rather controversial process (Gehring, 2010). Until a few years ago a *Z. mays* protein that participates in pollen tube growth and reorientation has remained the only experimentally confirmed AC in plants (Moutinho et al., 2001). In order to systematically identify candidate ACs in *A. thaliana*, we decided to undertake searches with modified GC motifs (**Figure 2**) since the aa in position 3 confers substrate specificity (Tucker et al., 1998; Roelofs et al., 2001; Wong and Gehring, 2013a). In GCs the residues in position 3 are [C, T, G, or H] whereas in ACs they are [D] or [E] (**Figure 2**). We then queried the Arabidopsis proteome using this substituted motif ([RKS][YFW][DE][VIL]X(8,9)[KR]X(1,3)[DE]) and retrieved 341 candidates proteins (Al-Younis et al., 2015). We then narrowed the search by including an N-terminal [R] 5–20 aa upstream of position 1 since [R] is essential for pyrophosphate-binding (Liu et al., 1997). This extended AC motif identifies 14 candidates. Testing of AC activity is somewhat more convenient than testing for GC activity since one can make use of an *Escherichia coli* cyaA mutant SP850 strain deficient in the AC (cyaA) gene (Shah and Peterkofsky, 1991) for rescue-based selection. Several of the candidates have since been tested and shown to be capable to rescue the cyaA mutant. One of them is the *A. thaliana* K^+^-uptake permease 7 (AtKUP7, At5g09400). In AtKUP7, an AC catalytic center containing cytosolic fragment has also been shown with tandem MS methods to generate cAMP from ATP (Al-Younis et al., 2015). The biological role of this molecule remains to be elucidated since it might act as a cAMP-dependent K^+^-flux sensor and is still under investigation.

It is noteworthy that in Paramecium, cAMP formation has been reported to be linked to K^+^ conductance, and this conductance is an intrinsic property of the AC (Weber et al., 2004). This multi-domain AC therefore functions as both an AC and a K^+^ channel in which a S4 voltage-sensor is part of the N-terminus and a K^+^ pore-loop occupies the C-terminus on the cytoplasmic side (Weber et al., 2004). AtKUP7 also has such dual domain architecture and may function as both K^+^ transporter and an AC; however, AtKUP7 is likely to be a proton-coupled carrier rather than a K channel. The future will tell if cAMP production is dependent on K^+^ fluxes and/or if cAMP can modulate K^+^ fluxes.

## Predicting Novel ACS and GCS with the Support of Structural Modeling

Molecular methods for the study of signal transduction have steadily developed together with technical advances (Irving and Gehring, 2013) and this is also reflected in the discovery and characterization of cNMPs and their cyclases. These advances include computational methods for large-scale sequence analyses of the ever-increasing number of fully sequenced (plant) genomes in the public domain and the growing number of published structures of MNCs, ACs, and GCs. These two resources have indeed allowed a computational approach to the identification of candidate MNCs in higher plants (Wong and Gehring, 2013b). A method to achieve accurate predictions of Arabidopsis candidate MNCs has been detailed and includes pattern searches with catalytic center motifs of ACs or GCs. It is also recommended that these centers of candidate MNCs are compared with orthologs of closely as well as more distantly related species since conservation, particularly in the key residues, to add confidence to the predication.

An example are the systemically mobile peptidic plant hormones (the PNPs) that elicit many physiological responses at nanomolar concentrations (Ludidi et al., 2002; Maryani et al., 2003; Gottig et al., 2008; Gehring and Irving, 2013; Turek et al., 2014). The *A. thaliana* PNP-A (At2g18660) is a ligand of AtPNP-R1 (At1g33612) receptor (Turek and Gehring, 2016) that we have shown to be a ligand-activated receptor kinase with GC activity. The ortholog in tomato is a protein (K4D0Y5_SOLLC) that also contains the key residues essential for catalysis. And given that a Verticillium natriuretic peptide-like molecule is the ligand for the tomato Verticillium wilt immune receptor (C4NAS0| C4NAS0_SOLLC Verticillium wilt disease resistance protein), it does not come as a surprise that this receptor also comes with a GC motif and may conceivably be a GC as well (de Jonge et al., 2012).

Furthermore, it has also proven useful to subject candidate MNCs to structural modeling against structure templates of annotated MNCs and in addition, docking of the substrate, ATP or GTP, to ascertain if the catalytic center forms a cavity with the key residues for ATP of GTP interaction and the metal-binding residue [an example is shown in Wong and Gehring (2013a)]. Incidentally, such an approach can also be used to model mutations and predict if candidates are conceivably functional as cyclases (Wong et al., 2015).

## In Search of Novel Biological Functions of CNMPS and MNCS

Systems-based approaches to biology have also contributed and continue to contribute to or understanding of the breadth and complexity of cNMP-dependent cellular processes. The cGMP-dependent signals and their role in phosphorylation was examined using a phosphoproteomics approach and it was shown that upon treatment of *A. thaliana* roots with membrane-permeable cGMP a number of plant hormone-dependent microsomal proteins changed their phosphorylation status thus linking plant hormone responses to hormone-dependent cGMP transients and cGMP-dependent downstream phosphorylation (Isner et al., 2012). More recently a quantitative cGMP-dependent phosphoproteome of *A. thaliana* suspension-cultured cells that were metabolically labeled with ^15^N has revealed highly specific responses and points to hitherto unknown cGMP-dependent phosphorylation and dephosphorylation events (Marondedze et al., 2016a). A pathway analysis of the identified proteins revealed that seven of them are part of the spliceosome (Matera and Wang, 2014) and belong to four different spliceosome complexes. They include the pre-mRNA processing protein (At1g44910) and splicing factor PW1 (At1g60200) that belong to the U1 complex; the WD40 repeat protein (At2g32700) is part of the U4/U6 complex; the periodic tryptophan protein 2 (At1g15440, AtPWP2) belongs to the U5 complex; the UBP1-associated protein 2A (At3g56860), the RNA-binding protein 47C (At1g47500), and the R/S-rich splicing factor that are common spliceosome components. There is firm evidence that the alternative splicing and hence the spliceosome have an important role in biotic and abiotic stresses responses and that mutations in splicing factors and spliceosomal proteins are predicted to modulate the circadian clock and the highly complex plant defense responses (e.g., Staiger and Brown, 2013) and that the phosphorylation status of spliceosome components can affect splicing (Feng et al., 2008; Lipp et al., 2015) including in responses to stimuli. This points to a direct or indirect role of cGMP in splicing-mediated stress responses.

A recent study has used an affinity purification approach to identify cyclic nucleotide-binding proteins in *A. thaliana* (Donaldson et al., 2016). Of the 15 novel candidate cNMP-binding proteins (**Table 1**), several have key functions in the Calvin cycle and photorespiration pathway, and 8 of the 15 were shown to contain cyclic nucleotide-binding domains. What is particularly interesting is the fact that the identified proteins are post-translationally modified by NO, co-expressed, and annotated to function in responses to H_2_O_2_ and defense response (Donaldson et al., 2016). The activity of one of cGMP interacting proteins, glycolate oxidase 1 (At3g14420) is a photorespiratory enzyme that produces H_2_O_2_ in response to Pseudomonas, and was shown to be repressed by the combination of cGMP and NO treatment. This gave rise to the hypothesis that these cNMP-binding proteins (co-)operate as points of cross-talk between cyclic nucleotides, NO, and ROS signaling during defense responses (Donaldson et al., 2016).

**Table 1:** List of candidate cNMP interacting proteins.

Accession	Annotation	GO categories
At1g09340	Chloroplast RNA (stem loop)-binding protein, CRB	1, 2, 3, 4, 5
At1g42970	Glyceraldehyde-3-phosphate dehydrogenase b subunit	2, 3, 4, 5
At1g56330	Secretion-associated Ras 1b; GTP-binding protein	1
At3g01500	β-Carbonic anhydrase βCA1; salicylic acid-binding	1, 2, 3, 4, 5
At3g12780	Nuclear phosphoglycerate kinase, PGK1	1, 2, 3, 4
At3g13920	Eukaryotic translation initiation factor 4A1	
At3g14420	Glycolate oxidase 1, GOX1	5
At3g60750	Transketolase, TKL1	3, 4, 5
At3g62560	Ras-related small GTP-binding family protein	
At4g02080	A member of ARF-like GTPase family	
At4g09650	ATP synthase δ-subunit; pigment defective	2, 3, 4, 5
At4g20360	RAB GTPase homolog E1B	
At4g27440	Light-dep. NADPH:protochlorophyllide oxidoreductase B	5
At4g37930	Serine hydroxymethyltransferase 1	1, 2, 3, 4
At5g50920	ATP-dependent Clp protease	4, 5

*The GO analysis was performed in: http://bioinfo.cau.edu.cn/agriGO/. 1, GO:0009409: response to cold (FDA 6.3e^-*08*^); 2, GO:0009266: response to temperature stimulus (FDA 3.1e^-*07*^); 3, GO:0009628: response to abiotic stimulus (FDA 6.6e^-*06*^); 4, GO:0009526: plastid envelope (FDA 8.5e^-*12*^); and 5, GO:0009507: chloroplast (FDA 1.9e^-*05*^).*

## Out-Look

The discovery of an increasing number of plant MNCs does suggest that cAMP and cGMP have critical and complex roles in plant development, physiology, and responses to the environment. This is also supported by the diversity of the domain organizations of the MNCs themselves, which include NO sensors, receptor kinases, and ion channels. Not only do these “moonlighting” MNCs have to be characterized at the single molecule level, it will also be necessary to use genetic tools to elucidate their biological functions and in particular the role the cNMPs play in the downstream responses. There are already indications that the cNMPs act together with other messengers such as Ca^2+^. We can also expect to obtain further information on cNMP-dependent effects through investigations at the systems level, in particular extensive studies on cAMP- and cGMP-specific phosphorylation events, and the effect of the nucleotides on the metabolome. The recent report on Arabidopsis RNA-binding proteome has revealed that >20 RNA-binding proteins are spliceosome components (Marondedze et al., 2016b) and it will therefore be interesting to see if stress responses affect the RNA-binding proteome particularly since cGMP has been shown to cause specific cGMP-dependent phosphorylation of spliceosome components (Marondedze et al., 2016a).

In addition, there is increasing evidence that not only cAMP and cGMP function as messengers or modulators but also non-canonical nucleotides such as cCMP or cIMP. These molecules have long been known to be present in plants (Newton et al., 1999), and it has recently been demonstrated that particularly cIMP can induce significant ROS production. Incidentally, when homology models of experimentally confirmed plant GCs were probed with substrates other than GTP, it appears conceivable that they might function (Marondedze et al., 2017). This is of course not answering the question if there are cyclases that are specific for cCMP or cIMP.

A possibly the most intriguing unanswered question is where the plant 3′,5′-cyclic-nucleotide phosphodiesterases (PDEs) in higher plants are. Again, much like in the case of the cyclases, a BLAST search with an annotated *C. reinhardtii* PDE (A8HNW2_CHLRE) does not return any plausible candidate with reasonable similarity. However, recently a liverwort (*Marchantia polymorpha*) protein has been reported that has both AC and PDE domain and both activities (Kasahara et al., 2016). Incidentally, the molecule does contain an AC catalytic center motif ([RKS]X[CTGH]X{8,12}[KR]X{1,3}[DE]) but has no ortholog in Arabidopsis or other higher plants for that matter.

## Author Contributions

Both authors have made a substantial, direct and intellectual contribution to the work, and approved it for publication.

## Conflict of Interest Statement

The authors declare that the research was conducted in the absence of any commercial or financial relationships that could be construed as a potential conflict of interest.
